# Glycolysis Inhibition Inactivates ABC Transporters to Restore Drug Sensitivity in Malignant Cells

**DOI:** 10.1371/journal.pone.0027222

**Published:** 2011-11-02

**Authors:** Ayako Nakano, Daisuke Tsuji, Hirokazu Miki, Qu Cui, Salah Mohamed El Sayed, Akishige Ikegame, Asuka Oda, Hiroe Amou, Shingen Nakamura, Takeshi Harada, Shiro Fujii, Kumiko Kagawa, Kyoko Takeuchi, Akira Sakai, Shuji Ozaki, Kazuma Okano, Takahiro Nakamura, Kohji Itoh, Toshio Matsumoto, Masahiro Abe

**Affiliations:** 1 Department of Medicine and Bioregulatory Sciences, University of Tokushima Graduate School of Medicine, Tokushima, Japan; 2 Department of Medicinal Biotechnology, Institute for Medicinal Research, University of Tokushima Graduate School of Pharmaceutical Sciences, Tokushima, Japan; 3 Department of Pediatrics, University of Tokushima Graduate School of Medicine, Tokushima, Japan; 4 Division of Transfusion Medicine, Tokushima University Hospital, Tokushima, Japan; 5 Department of Hematology and Oncology, RIRBM, Hiroshima University, Hiroshima, Japan; 6 Division of Internal Medicine, Tokushima Prefectural Hospital, Tokushima, Japan; University of Nebraska – Lincoln, United States of America

## Abstract

Cancer cells eventually acquire drug resistance largely via the aberrant expression of ATP-binding cassette (ABC) transporters, ATP-dependent efflux pumps. Because cancer cells produce ATP mostly through glycolysis, in the present study we explored the effects of inhibiting glycolysis on the ABC transporter function and drug sensitivity of malignant cells. Inhibition of glycolysis by 3-bromopyruvate (3BrPA) suppressed ATP production in malignant cells, and restored the retention of daunorubicin or mitoxantrone in ABC transporter-expressing, RPMI8226 (ABCG2), KG-1 (ABCB1) and HepG2 cells (ABCB1 and ABCG2). Interestingly, although side population (SP) cells isolated from RPMI8226 cells exhibited higher levels of glycolysis with an increased expression of genes involved in the glycolytic pathway, 3BrPA abolished Hoechst 33342 exclusion in SP cells. 3BrPA also disrupted clonogenic capacity in malignant cell lines including RPMI8226, KG-1, and HepG2. Furthermore, 3BrPA restored cytotoxic effects of daunorubicin and doxorubicin on KG-1 and RPMI8226 cells, and markedly suppressed subcutaneous tumor growth in combination with doxorubicin in RPMI8226-implanted mice. These results collectively suggest that the inhibition of glycolysis is able to overcome drug resistance in ABC transporter-expressing malignant cells through the inactivation of ABC transporters and impairment of SP cells with enhanced glycolysis as well as clonogenic cells.

## Introduction

The emergence of drug-resistant clones during the course of treatment and the presence of cancer stem cells or cancer-initiating cells are among the predominant causes of drug resistance in cancer patients [1], [2]. Such drug-resistant cells increase their expression of ATP-binding cassette (ABC) transporters including P-glycoprotein (ABCB1), breast cancer resistance protein (BCRP; ABCG2) and multidrug-resistance-associated protein-1 (MRP-1), which function as efflux transporters dependent on energy from the hydrolysis of ATP for a variety of chemotherapeutic drugs [1], [2], [3], [4], [5]. Cancer stem cells or cancer-initiating cells have a tumor-initiating capacity and appear to be involved in resistance to chemotherapy and tumor relapse [1], [2]. They are considered to be contained in a “side population” with negative staining of Hoechst 33342 fluorescence dye, a substrate for BCRP, suggesting higher ABC transporter activity in these cells [6], [7], [8], [9].

Malignant cells increase their expression of glycolytic enzymes and glucose uptake to markedly enhance glycolysis (aerobic glycolysis; the Warburg effect), which leads to the production of a large amount of ATP and biomass such as nucleic acids and lipids essential for cell survival and division [10], [11], [12]. Thus, increased aerobic glycolysis is regarded as a hallmark of cancers and applied to the detection of malignant lesions in [^18^F]fluorodeoxyglucose-positron emission tomography (FDG-PET) which is widely used in clinics [12], [13]. In parallel with enhanced glycolysis, ATP production by oxidative phospohorylation in the tricarboxylic acid (TCA) cycle in mitochondria is suppressed through oncogenic alterations including the mutation of p53 [10], [12], [14]. In sharp contrast to malignant cells with glycolysis-dependent ATP production, normal cells utilize the TCA cycle in mitochondria for their ATP [10], [11], [12], [14], [15]. These observations suggest that the inhibition of glycolysis can abolish ATP production as well as biomass synthesis in cancer cells while sparing ATP production and cell metabolism in normal quiescent cells; and thus enhanced glycolysis may become a novel cancer-specific target for anti-cancer treatment.

Drug resistance has emerged as an important clinical issue in the treatment of cancers; and ABC transporters are regarded as a major target in drug-resistant cancer cells. Because ABC transporter activity is dependent on ATP [5], [16] and because ATP production in cancer cells is largely dependent on enhanced glycolysis [10], [11], [12], [14], [15], we hypothesized that inhibition of glycolysis can induce a cancer-specific inactivation of ABC transporter activity to restore susceptibility to anti-cancer drugs. We demonstrate herein that inhibition of glycolysis preferentially targets malignant cells to suppress ATP production, and that inhibition of glycolysis inactivates ABC transporter activity to retain anti-cancer agents intracellularly and restore their cytotoxic effects on malignant cells.

## Materials and Methods

### Ethics Statement

All procedures involving human specimens were performed with written informed consent according to the Declaration of Helsinki and using a protocol approved by the Institutional Review Board for human protection in University of Tokushima (Permit number: 240). The mouse experiment was carried out in strict accordance with the recommendations in the Guide for the Care and Use of Laboratory Animals of the National Institutes of Health. The protocol was approved by the Animal Experimentation Committee of the University of Tokushima (Permit number: 10120). All efforts were made to minimize suffering.

### Reagents

The following reagents were purchased as indicated: 3BrPA and verapamil from Sigma (St. Louis, MO); mouse monoclonal anti-human BCRP from Millipore (Temecula, CA); mouse monoclonal anti-human MRP1 from Santa Cruz Biotechnology (Santa Cruz, CA); FITC-rabbit anti-mouse IgG from Zymed Laboratories (San Francisco, CA); and PE-mouse anti-P-glycoprotein antibody, PE-mouse anti-human CD138 antibody, and PE-mouse IgG from BD Bioscience (San Jose, CA).

### Cells and cultures

The human KG1 leukemic cell line, RPMI8226 myeloma (MM) cell line, HepG2 hepatoma cell line, DU145 prostate carcinoma cell line, and MDA-MB231 breast cancer cell lines were obtained from American Type Culture Collection (ATCC) (Rockville, MD). The MM cell line INA6 was kindly provided by Dr. Renate Burger (University of Kiel, Kiel, Germany). Bone marrow mononuclear cells (BMMCs) were isolated from fresh bone marrow aspirates of patients with myeloma and primary CD138^+^ myeloma cells were further sorted using CD138 MicroBeads (Miltenyi Biotec, Bergisch Gladbach, Germany) as described previously [17], [18]. Peripheral blood mononuclear cells (PBMCs) were isolated from fresh peripheral blood from healthy donors [18]. KG1 and RPMI8226 cells and primary hematopoietic cells were cultured in RPMI1640 medium (Sigma) supplemented with 10% fetal bovine serum (FBS), 100 U/ml of penicillin G, and 100 µg/ml of streptomycin (Sigma). INA-6 cells were cultured in RPMI1640 medium supplemented with 10% FBS, 100 U/ml of penicillin G, 100 µg/ml of streptomycin, and 1 ng/ml of rhIL-6 (PEPROTECH EC, London, UK). HepG2, DU145, and MDA-MB231 cells were cultured in αMEM (Sigma) supplemented with 10% FBS, 2 mM L-glutamine (Sigma), 100 U/ml of penicillin G, and 100 µg/ml of streptomycin.

### Colony formation assays

Cells were cultured in duplicate at 200–500 cells/ml in 35 mm dishes containing IMDM (Sigma) with 1.17% of methylcellurose (R&D Systems), 30% of FBS, 100 U/ml of penicillin G, and 100 µg/ml of streptomycin. After culturing for about 2 weeks, colonies were visualized and counted under an Olympus BX50 microscope equipped with an UMPlanFI 40X/0.75 objective lens (Olympus, Tokyo, Japan). Images were recorded with an Olympus SC35 CCD camera and Viewfinder Lite Software (Pixera, Los Gatos, CA).

### Cell viability assays

Cells were plated out in triplicate in 96-well culture plate and incubated with drugs. The number of viable cells was determined by the Cell Counting Kit-8 assay (DOJINDO, Kumamoto, Japan) according to the manufacturer's instructions. The absorbance of each well was measured at 450 nm with a microplate reader (Model 450 micro plate reader; Bio-Rad Laboratories, Hercules, CA). Data was shown as the mean+- SD.

### Intracellular ATP measurements

Cells were plated in duplicate in 96-well culture plates. Cellular ATP levels were determined using the CellTiter-Glo luminescent Cell Viability Assay (Promega, Madison, USA) according to the manufacturer's instructions. Luminescent levels were measured by microplate reader Thermo (Thermo Fisher Varioskan Flash, Waltham MA).

### Flow cytometry

Cells were collected and stained with PE-labeled mouse monoclonal anti-P-glycoprotein antibody, or with mouse monoclonal anti-BCRP or anti-MRP1 antibody followed by FITC-labeled rabbit anti-mouse IgG antibody as described before [19]. The expression of P-glycoprotein, BCRP, and MRP1 was determined using Coulter Epics XL-MCL (Beckman & Coulter) and analyzed by CellQuest software (BD Bioscience). For analysis of the CD138^+^ population, BMMCs were stained with FITC-labeled mouse anti-CD138 antibody and the population of CD138^+^ myeloma cells was analyzed by flow cytometry. Apoptosis was evaluated by staining cells with an annexin V-FITC and propidium iodide (PI) labeling kit (MEBCYTO Apoptosis Kit; MBL,Nagano, Japan) according to the manufacturer's instructions.

### Drug accumulation and efflux assay

Cells were cultured in the presence or absence of 3BrPA, and 30 minutes later, 30–100 ng/ml of daunorubicin or 100 ng/ml of mitoxantrone was added. After incubating for 30 minutes, cells were washed, and intracellular fluorescence levels were analyzed by flow cytometry (accumulation phase, AP). For determining drug efflux levels, the cells were further incubated in the presence or absence of 3BrPA for 120 minutes, and intracellular fluorescence levels were analyzed (efflux phase, EP). Intracellular drug concentrations at AP and EP were represented as ΔMFI_AP_ (the difference of MFI between AP and background) and ΔMFI_EP_ (the difference of MFI between EP and background), respectively.

### Lactate measurements

After filtering culture media to remove protein, lactate levels were measured using Lactate Assay Kit (BioVision, Mountain View, CA). The absorbance of each well was measured at 570 nm with a microplate reader (Model 450 micro plate reader).

### Side population (SP) analysis

Cells were incubated with 5 µg/ml of Hoechst 33342 (Invitrogen) for 90 min at 37 °C in the presence or absence of 100 µM of verapamil or 3BrPA. After being washed, the cells were resuspended in ice-cold PBS supplemented with 1 µg/ml of PI to detect dead cells. Hoechst 33342 was excited with a UV laser at 350 nm, and the SP and main population (MP) cell fractions were analyzed by flow cytometry (EPICS ALTRA HyperSort, Beckman & Coulter) with 450 nm (Hoechst blue) and 675 nm (Hoechst red) filters. The SP and MP fractions were sorted by flow cytometry.

### Quantitative real-time PCR

Cells were harvested and total RNA was extracted from cells using TRIZOL reagent (Invitrogen Life Technologies, Carlsbad, CA). Equal amounts of total RNA were subjected to reverse transcription using Superscript II (Invitrogen). Real-time PCR was performed using Platinum SYBR Green qPCR SuperMix UDG with Rox (invitrogen) with the following amplification program: one cycle of 50 °C for 2 minutes and 95 °C for 2 minutes and 40 cycle of 95 °C for 15 seconds and 60 °C for 30 seconds. The reaction was followed by a melting curve protocol according to the specifications of the ABI 7300 (Applied Biosystems, Foster City, CA). Primers used were as follows: h*HKII* sense 5′-TGGAGGGACCAACTTCCGTGTGCT-3′ and antisense 5′-TCAAACAGCTGGGTGCCACTGC-3′, h*GAPDH* sense 5′-AATCCCATCACCATCTTCCA-3′ and antisense 5′-TGGACTCCACGACGTACTCA-3′, h*PDK1* sense 5′-CGGATCAGAAACCGACACA-3′ and antisense 5′-GGATCAGAAACCGACACA-3′, h*PKM2* sense 5′-TCAAGTGCTGCAGTGGGGCCAT-3′ and antisense 5′-TCACAGCAATGATGGGGGCACGT-3′, h*PFK2* sense 5′-TGTCGCTTATGGCTGCCGTGT-3′ and antisense 5′-AGCGGGGTGACACTATTGCGT-3′, and h*HPRT1* (used as a housekeeping gene for normalization) sense 5′-TTTGCTTTCCTTGGTCAGGC-3′ and antisense 5′-GCTTGCGACCTTGACCATCT-3′.

### Immunoblotting

Cells were harvested and fractioned into cytosolic and membrane fractions using a Mem-PER Eukaryotic Membrane Protein Extraction Kit (Thermo Scientific, Rockford, IL). Membrane fractions were subjected to Immunoblotting analysis as described previously [18]. Apoptosis inducing factor (AIF) was used as a loading control of membrane protein.

### In vivo experiments

Five-week-old male SCID mice (CLEA Japan, Tokyo, Japan) were injected intraperitoneally with 100 µg of rabbit anti-acialo-GM1 antibody (Wako, Osaka, Japan) for the inactivation of NK cells one day before the subcutaneous inoculation of RPMI8226 cells (1×10^6^). After confirmation that tumors over 100 mm^3^ has formed, the mice were intraperitoneally injected 3 times a week with 200 µl of PBS, DOX (0.5 mg/kg), 3BrPA (5mg/kg), or DOX plus 3BrPA. Tumor volume and body weight were measured before each injection. Tumor volume was calculated as length × width × height/2.

### Statistical analysis

Data are represented as means +/− standard deviations unless specified otherwise. Statistical significance was determined by a one-way analysis of variance (ANOVA) with Scheffe's post hoc tests or Student's t-test. The minimal level of significance was P = 0.05.

## Results

### Inhibition of glycolysis by 3BrPA reduces ATP production and induces cell death preferentially in malignant cells

**Figure 1:**
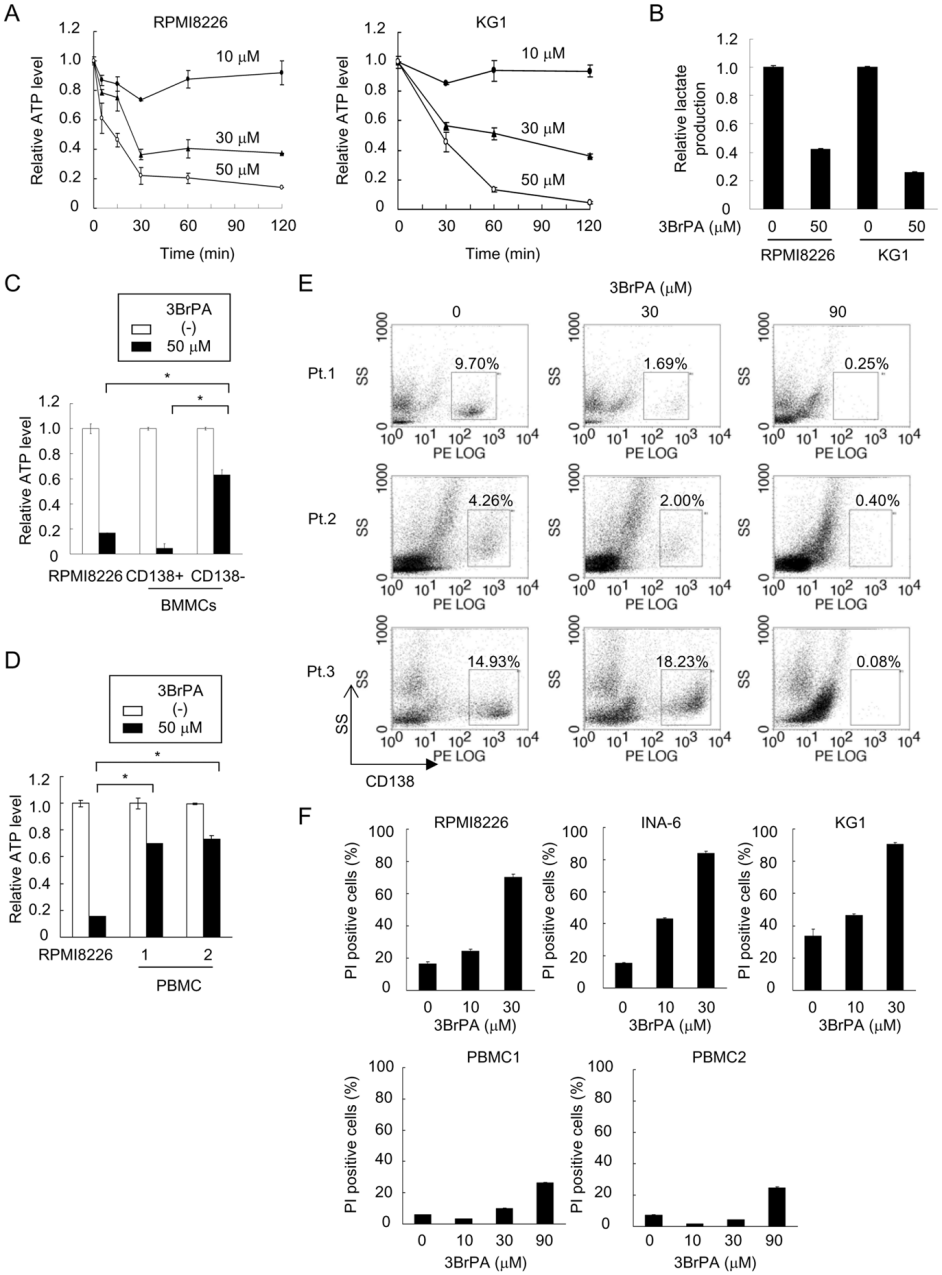
Reduction in ATP production and viability of malignant cells by 3BrPA. **A.** RPMI8226 and KG1 cells were cultured with 3BrPA at 10, 30 and 50 µM. Cellular levels of ATP were measured at different time points as indicated. **B.** RPMI8226 and KG1 cells were cultured for 2 hours in the absence or presence of 3BrPA at 50 µM. The levels of lactate in their culture supernatants were measured. **C, D.** CD138^+^ primary MM cells and CD138^-^ non-MM BMMCs cells or PBMCs were cultured for 60 minutes with 50 µM of 3BrPA, and cellular levels of ATP were measured. Data were expressed relative values for untreated cells. *, P<0.05 **E.** BMMCs from patients with MM were cultured for 3 days with 30 and 90 µM of 3BrPA. The cells were then stained with PE-labeled anti-CD138 antibody, and analyzed by flow cytometry. Cell distributions were analyzed by the intensity of side scatter (ss) vs. CD138 levels, and rectangles contained CD138^+^ MM cells. **F.** RPMI8226, INA-6 and KG1 cells, and PBMCs were cultured for 24 hours with 3BrPA at the indicated concentrations. The cells were stained with PI for and analyzed by flow cytometry.

Malignant cells have been demonstrated to produce a large amount of ATP through glycolysis [10], [11], [12], [14], [15]. In order to clarify the role of glycolysis in ATP production in malignant and normal cells, we first measured the intracellular levels of ATP upon treatment with the hexokinase II inhibitor 3BrPA, a potent glycolysis inhibitor, in malignant cells. ATP levels in RPMI8226 multiple myeloma (MM) cells were substantially decreased by 3BrPA at 30 µM in as little as 30 minutes (Figure 1A). The effects were dose-dependent and persisted at the same levels for 2 hours. 3BrPA similarly suppressed ATP production in KG-1 leukemic cells (Figure 1A). Because the levels of lactate production reflect glycolytic activity, we also measured the levels of lactate produced by RPMI8226 and KG1 cells to determine glycolytic activity. Addition of 3BrPA reduced the lactate production in these cells (Figure 1B), confirming the inhibition of glycolysis by 3BrPA.

MM remains essentially incurable by conventional treatment, and represents a drug-resistant hematological malignany [20], [21]. MM cells reside in the bone marrow with normal hematopoietic cells. To compare the effects of inhibiting glycolysis on ATP production between malignant and normal cells, we isolated CD138^+^ primary MM cells and CD138^-^ non-MM cells from bone marrow samples of patients with MM and PBMCs from normal subjects. Treatment with 3BrPA at 50 µM suppressed ATP production markedly in CD138^+^ primary MM cells as in RPMI8226 cells but only partially in normal CD138^-^ bone marrow cells and PBMCs (Figure 1C–D). These results are consistent with the notion that ATP production is dependent on glycolysis in malignant cells. We next examined whether the depletion of ATP causes tumor-specific impairment of cell viability. 3BrPA dose-dependently reduced CD138^+^ MM cell fractions in bone marrow mononuclear cells from patients with MM (Figure 1E). Of note, after treatment with 3BrPA at 90 µM, the MM fractions were almost extinguished while remaining non-MM bone marrow cells. 3BrPA at 30 µM also induced cell death in RPMI8226 and INA6 MM and KG1 leukemic cells but marginally in normal PBMCs (Figure 1F). These results suggest that inhibition of glycolysis preferentially targets malignant cells to suppress ATP production and induce cell death.

### Inhibition of glycolysis by 3BrPA enhances drug accumulation and retention in ABC transporter-expressing malignant cells

**Figure 2:**
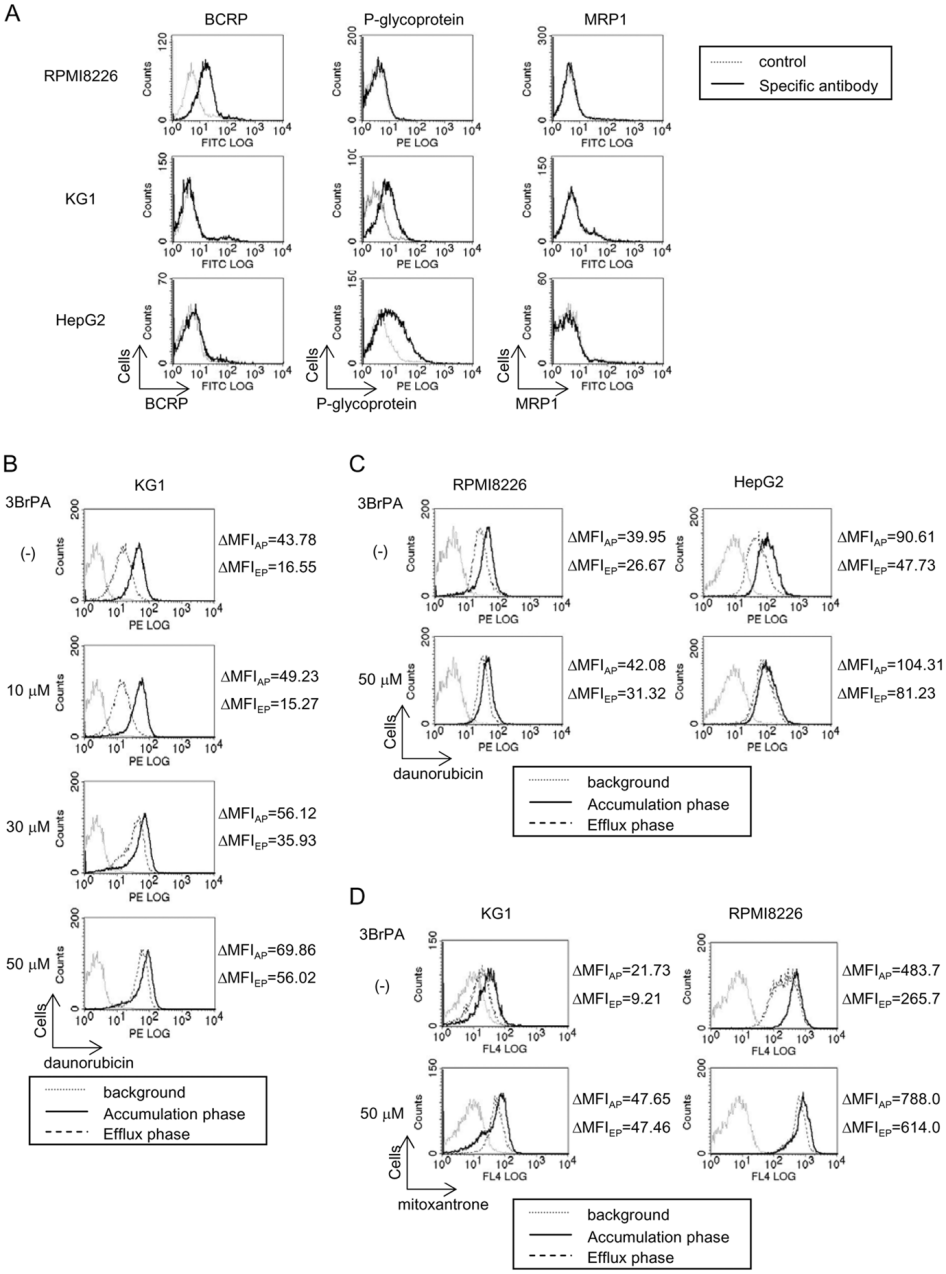
Inactivation of ABC transporter activity in malignant cells by 3BrPA. **A.** Surface expression of ABC transporters in KG1, RPMI8226 and HepG2 cells. **B.** KG1 cells were cultured with 3BrPA at the indicated concentrations. After 30 minutes, daunorubicin was added and the cultures incubated for 30 minutes (accumulation phase, AP). The cells were then washed and further incubated for 2 hours in medium without daunorubicin in the presence of 3BrPA at the indicated concentrations (efflux phase, EP). **C, D.** Fluorescein intensity was measured by flow cytometry to represent intracellular levels of daunorubicin. The intracellular retention of daunorubicin in RPMI8226 and HepG2 cells and mitoxantrone in KG1 and RPMI8226 cells was similarly analyzed.

To examine whether the ABC transporter activity in malignant cells depends on ATP produced by enhanced glycolysis, we first looked for ABC transporter-expressing malignant cells. After screening, we obtained three malignant cell lines with high ABC transporter expression: RPMI8226 MM and KG-1 leukemic cells constitutively over-expressed BCRP and P-glycoprotein, respectively (Figure 2A), while HepG2 hepatoma cells weakly expressed BCRP and strongly expressed P-glycoprotein.

We next examined the effects of inhibition of glycolysis on ABC transporter activity in these ABC transporter-expressing cells. ABC transporter activity was determined based on the intracellular accumulation and retention of daunorubicin and mitoxantrone, auto-fluorescence emitting anti-cancer agents known as a substrate for ABC transporters [22], [23]. KG1 cells pretreated with 3BrPA were further incubated with daunorubicin for 30 minutes to passively incorporate it into the cells. 3BrPA dose-dependently increased intracellular daunorubicin levels after the 30-minute drug accumulation phase (Figure 2B). To analyze the intracellular retention of the drug, the cells were washed and incubated for 2 hours in medium without daunorubicin. After the incubation, the cellular content of daunorubicin was markedly reduced in the absence of 3BrPA as observed by MIF levels from 43.78 to 16.55 (Figure 2B). However, most of the daunorubicin was retained in KG-1 cells in the presence of 3BrPA at 30 and 50 µM, concentrations high enough to reduce intracellular ATP levels as shown in Fig. 1B. These results suggest that 3BrPA is able to effectively accumulate and retain daunorubicin in ABC transporter-expressing malignant cells. The potential use of 3BrPA for intracellular drug accumulation and retention was further studied with ABC transporter-expressing RPMI8226 and HepG2 cells (Figure 2C). 3BrPA at 50 µM enhanced the accumulation of daunorubicin and restored its retention in these cells as observed in KG-1 cells. Similar results were obtained with mitoxantrone in KG-1 and RPMI8226 cells (Figure 2D). Because surface levels of P-glycoprotein and BCRP were not changed in KG1 and RPMI8226 cells, respectively, after the treatment with 3BrPA (Figure S1), these results suggest that ABC transporter activity is dependent on glycolysis in malignant cells, and that depletion of intracellular ATP by inhibition of glycolysis is able to inactivate ABC transporters to retain anti-cancer agents in malignant cells.

### Inhibition of glycolysis by 3BrPA effectively suppresses ATP production and drug efflux function in SP cells

**Figure 3:**
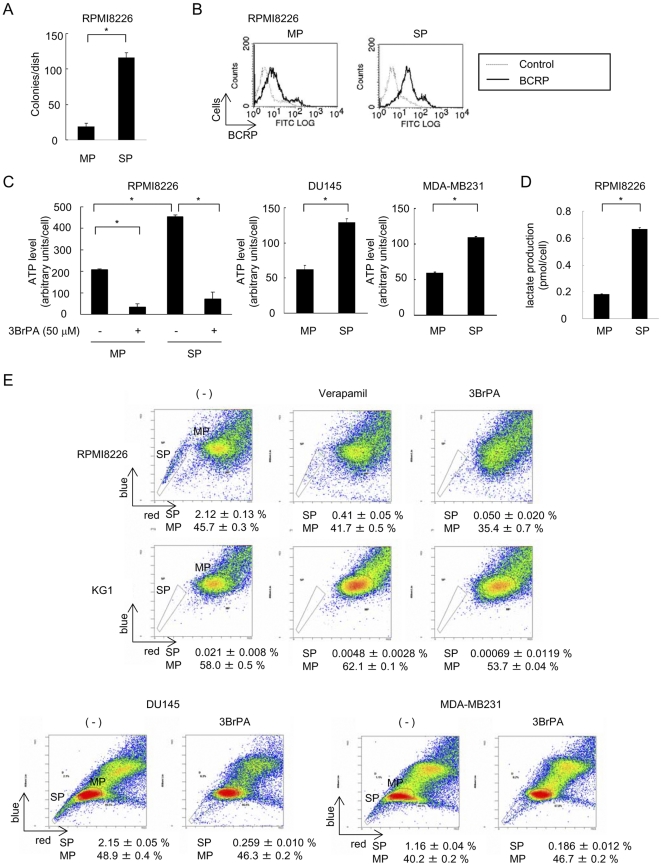
ATP production and drug efflux function in SP cells treated with 3BrPA. A. SP or MP cells were sorted from RPMI8226 cells, and cultured in medium containing methylcellulose. After 14 days, colonies were counted. **B.** Surface expression of BCRP in SP and MP cells. **C.** Cellular ATP levels in SP and MP cells. SP and MP cells from RPMI8226 cells were cultured for 60 minutes with 50 µM of 3BrPA, and then cellular ATP levels were measured. Cellular ATP levels were measured in SP and MP cells from DU145 and MDA-MB231 cells. *, P<0.05 **D.** SP and MP cells from RPMI8226 cells were cultured for 2 hours, and then lactate levels in their culture supernatants were measured. **E.** Substantial reduction of SP fractions by 3BrPA. RPMI8226, KG1, DU145, and MDA-MB231 cells were stained with Hoechst 33342 for 90 min in the presence of 3BrPA at 50 µM. Verapamil was added at 100 µM to determine SP cells, and distribution areas of SP cells were indicated.

Cell fractions with high ABC transporter activity are observed as a SP by Hoechst33342 dye staining which has been recognized in a variety of cancers [6], [7], [8], [9]. SP fractions are considered to contain cancer stem-like cells or cancer-initiating cells which are capable of self-renewal and play a critical role in drug resistance and relapse of tumors [1], [2]. Because of the clonogenic and proliferative capacity of SP cells, we assumed that glycolysis might be further enhanced in SP fractions in malignant cells to supply ATP and biomass to potentiate their growth and that inhibition of glycolysis could be an effective way of targeting SP cells. Because SP fractions were clearly observed in RPMI8226, DU145 prostate cancer, and MDA-MB231 breast cancer cell lines, we sorted SP and MP cells from these cells, and examined the status of glycolysis in these fractions and the impact of glycolysis inhibition on ATP production. We first looked at clonogenic capacity using colony formation in a semi-solid medium. Colony formation was enhanced about 6-fold in SP cells isolated from RPMI8226 cells compared to in MP cells (Figure 3A), confirming the clonogenic capacity of the SP cells. Surface levels of BCRP were also higher in the SP cells than MP cells (Figure 3B). SP cells isolated from RPMI8226 and MDA-MB231 cells exhibited an increased expression of genes involved in the glycolytic pathway including *GLUT1, GLUT3, PDK1* and *PFK2* (Figure S2). SP cells generated a larger amount of ATP per cell compared to MP cells in RPMI8226, DU145, and MDA-MB231 cells (Figure 3C). Lactate production per cell was also markedly increased in SP cells from RPMI8226 cells compared to that in MP cells (Figure 3D), which further corroborate the enhancement of glycolysis in SP fractions. These results suggest that glycolysis is highly accelerated in SP cells. However, 3BrPA at 50 µM mostly suppressed the ATP production in SP cells to levels similar to those in MP cells (Figure 3C).

As demonstrated by Hoechst33342 dye staining, SP cells are characterized as a cell population with enhanced ABC transporter activity. Interestingly, SP cells were hardly detected in RPMI8226 and KG1 cells after treatment with 3BrPA as observed upon treatment with verapamil, an inhibitor of ABC transporters (Figure 3E). Similar results were obtained in DU145 and MDA-MB231 non-hematopoietic cancer cell lines. From these observations, the inhibition of glycolysis is suggested to effectively inactivate the ABC transporter function enhanced in SP cells.

### Inhibition of glycolysis by 3BrPA abrogates the colony forming capacity of malignant cells

**Figure 4:**
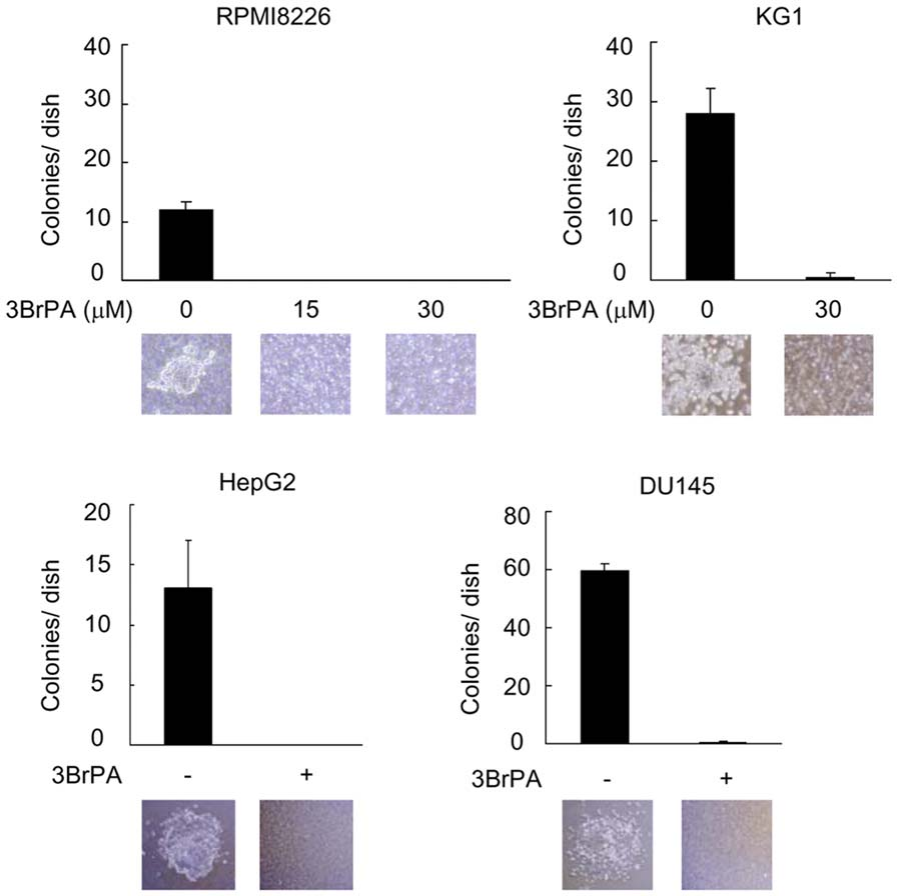
Inhibition of colony formation in malignant cells by 3BrPA. Cells were cultured in the presence of 3BrPA at the indicated concentrations in semi-solid metylcellulose medium. Colony numbers were counted after 2 weeks of culturing. Images of representative colonies formed in semi-solid medium were shown in the lower panels (x100, original magnification).

Given that glycolysis is highly enhanced in cells with tumor-initiating or progenitor potential in malignant tumors as observed in SP cells, the inhibition of glycolysis may enable the targeting of these cells to disrupt their clonogenic capacity. Therefore, we next examined the effects of 3BrPA on the formation of colonies by RPMI8226, KG1, HepG2, and DU145 cells. The ability of these cells to form colonies was strikingly reduced by 3BrPA (Figure 4). The results suggest that malignant cells with clonogenic capacity appear to be susceptible to damage through the inhibition of glycolysis.

### Inhibition of glycolysis by 3BrPA enhances the tumoricidal effects of anti-cancer agents on ABC transporter-expressing malignant cells

**Figure 5:**
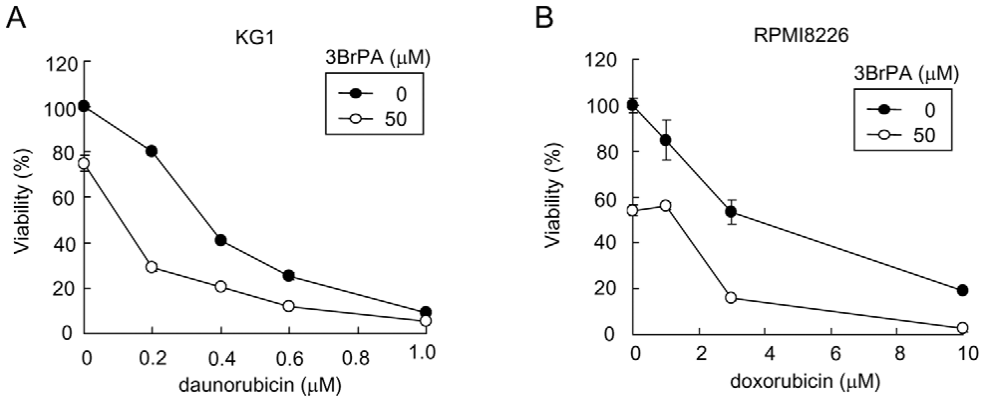
Enhancement of cytotoxic effects of anti-cancer agents by 3BrPA on ABC transporter-expressing malignant cells. **A, B.** KG1 and RPMI8226 cells were treated with daunorubicin for 60 minutes and doxorubicin for 90 minutes at the indicated concentrations, respectively, followed by 3BrPA. KG1 and RPMI8226 cells were then cultured with for a further 60 and 10 minutes, respectively, in the absence of 3BrPA or with 50 µM of 3BrPA. The cells were washed to remove 3BrPA and the drugs, and cultured for 22 hours in drug-free medium. Cell viability was determined by WST-8 assays.

**Figure 6:**
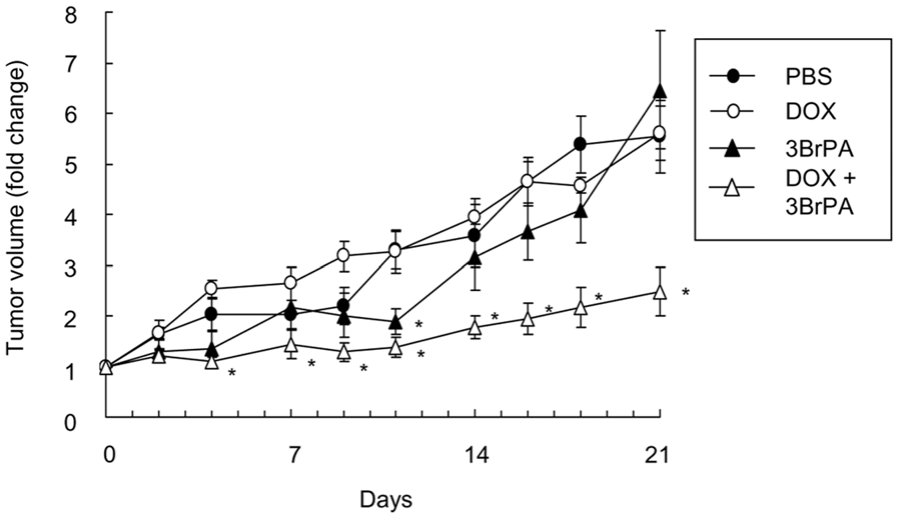
Suppression of tumor growth by 3BrPA and doxorubicin in vivo. RPMI8226 cells were subcutaneously inoculated into 5-week-old male SCID mice. After the formation of subcutaneous tumors over 100 mm^3^, the mice were treated 3 times a week for 3 weeks with either PBS, doxorubicin (0.5 mg/kg), 3BrPA (5mg/kg) or doxorubicin plus 3BrPA (n = 5 for each group). The results were expressed as fold increases in tumor volume from the baseline. *, P<0.05s.

Since the inhibition of glycolysis inactivates ABC transporters to retain anti-cancer agents in malignant cells (Figure 2B–C), we next examined the tumoricidal effects of anti-cancer agents in combination with 3BrPA in ABC transporter-expressing malignant cells. Exposure to daunorubicin dose-dependently induced cell death in KG-1 cells (Figure 5A). Co-treatment with 3BrPA substantially increased the cytotoxic effects of daunorubicin even at low concentrations, 0.2 and 0.4 µM, although 3BrPA alone only partially induced cell death in KG-1 cells. Similar results were obtained on the treatment of RPMI8226 cells with doxorubicin plus 3BrPA (Figure 5B). The effects of the combined treatment were further studied in vivo using SCID mice implanted subcutaneously with RPMI8226 cells. After confirmation that subcutaneous tumors had formed, mice were treated with doxorubicin, 3BrPA, or both. Doxorubicin or 3BrPA alone at these dosages had only marginal effects on tumor growth (Figure 6). However, in combination, they markedly suppressed subcutaneous tumor growth, indicating cooperative tumoricidal effects in vivo. The body weights of the mice did not change significantly during the course of treatment in any group (Figure S3). These results suggest that glycolysis inhibition restores the susceptibility of ABC transporter-expressing cells to chemotherapeutic agents.

## Discussion

Cancer cells eventually acquire drug resistance often through the aberrant expression of drug-expelling ABC transporters [2], [4]. Overexpression of ABC transporters prevents the sufficient accumulation of anticancer drugs within cells thereby confering drug resistance [22], [23]. Stem cell-like populations have been identified in a variety of hematopoietic and solid tumors [7], [8], [9]. Besides their clonogenic or tumor-initiating capacity, they exhibit high levels of ABC transporter expression and activity and confer resistance to drugs. Given the existence of cancer stem cells with ABC transporters, tumors can survive chemotherapy and eventually regrow. Therefore, ABC transporters are widely considered to be one of the most critical targets in the treatment of cancers, especially those with drug resistance. Various selective inhibitors of ABC transporters have been developed, and some are under clinical testing [24], [25], [26]. Most of them appear to compete with anti-cancer agents for the same binding site of certain ABC transporters [26], [27]. Other approaches include a molecular genetic strategy to selectively block the expression of individual ABC transporters using hammerhead ribozymes and antisense oligonucleotides [24], [25], [26]. However, drug-resistant cancer cells express multiple ABC transporters which act to expel drugs. Therefore, the efficacy of the above strategies targeting a single transporter appears to be limited. In contrast, inhibition of glycolysis is able to simultaneously inactivate all types of ABC transporters in cancer cells, because each transporter is dependent on ATP generated largely through enhanced glycolysis.

The mechanisms critical to the enhancement of glycolysis in cancer cells have been increasingly evident to be different from those in normal cells [28]. Certain isoforms of enzymes such as HKII [27], [29], [30] and PKM2 [31], [32] have been reported to be aberrantly up-regulated and responsible for the enhancement of glycolysis exclusively in cancer cells. Consistently, 3BrPA, a HKII inhibitor, preferentially acts on MM cells but marginally on normal cells (Figure 1C–F). In addition, 3BrPA markedly suppresses cell viability of clonogenic malignant cells (Figure 4) and inactivates ABC transporters in malignant cells including SP cells (Figure 2B–D, 3E). Although enhanced glycolysis is regarded as a characteristic feature of cancer cells [10], [12], [14], [15] and particularly SP fractions (Figure 3C–D, Figure S2), some types of normal cells increase glycolysis to maintain their metabolism in a context-dependent manner [11], [14]. In contrast to the constitutive enhancement of glycolysis in cancer cells in normal oxygen tension, hypoxia triggers to enhance glycolysis in normal cells. Hematopoietic stem cells have been demonstrated to reside within hypoxic areas in bone marrow and thus exhibit high glycolytic activity and low oxidative phosphorylation in the bone marrow [33], [34]. Although the inhibition of glycolysis appears to affect malignant cells but not normal quiescent mature hematopoietic cells (Figure 1C–E), further study is needed to evaluate the overall efficacy and toxicity of the inhibition of glycolysis in vivo.

The enhancement of glycolysis can also cause drug resistance in malignant cells through mechanisms other than the activation of ABC transporters. Acute lymphoblastic leukemia cells resistant to glucocorticoids have been reported to show aberrant change in the expression of glycolytic pathway-associated genes and increased glucose consumption compared with sensitive cells, and inhibition of glycolysis can resensitize the prednisolone-resistant leukemia cells with high metabolic activity without affecting the efflux of glucocorticoids [35]. Together with the present observations, inhibition of glycolysis is suggested to be able to effectively target drug-resistant and clonogenic tumor cells with a high metabolic state, and impair their metabolism to restore cytotoxic effects of anti-cancer agents. Therefore, these findings highlight a novel role for enhanced glycolysis in malignant cells in tumor growth and drug resistance, and relevance to anti-cancer strategies attempting to target cancer metabolism.

## Supporting Information

Figure S1 **Surface membrane ABC transporter levels by glycolysis inhibition. A.** RPMI8226 cells were cultured for 4 hours in the presence or absence of 3BrPA, and harvested. Membrane protein level of was analyzed by immunoblotting. **B.** Surface expression levels of P-glycoprotein in KG1 cells were analyzed by flow cytometry after culturing for 1 hour in the presence or absence of 3BrPA.
(TIF)

Figure S2 **Glycolytic gene expression.** Expression of genes involved in the glycolytic pathway were analyzed in SP and MP cells by quantitative real-time PCR.
(TIF)

Figure S3 **Body weight change.** Body weights of the RPMI8226-bearing mice were measured at every treatment.
(TIF)

## References

[1] Dean M, Fojo T, Bates S (2005). Tumour stem cells and drug resistance.. Nat Rev Cancer.

[2] Dean M (2009). ABC transporters, drug resistance, and cancer stem cells.. J Mammary Gland Biol Neoplasia.

[3] Doyle LA, Ross DD (2003). Multidrug resistance mediated by the breast cancer resistance protein BCRP (ABCG2).. Oncogene.

[4] Allen JD, Brinkhuis RF, van Deemter L, Wijnholds J, Schinkel AH (2000). Extensive contribution of the multidrug transporters P-glycoprotein and Mrp1 to basal drug resistance.. Cancer Res.

[5] Fletcher JI, Haber M, Henderson MJ, Norris MD (2010). ABC transporters in cancer: more than just drug efflux pumps.. Nat Rev Cancer.

[6] Wu C, Alman BA (2008). Side population cells in human cancers.. Cancer Lett.

[7] Haraguchi N, Utsunomiya T, Inoue H, Tanaka F, Mimori K (2006). Characterization of a side population of cancer cells from human gastrointestinal system.. Stem Cells.

[8] Ho MM, Ng AV, Lam S, Hung JY (2007). Side population in human lung cancer cell lines and tumors is enriched with stem-like cancer cells.. Cancer Res.

[9] Hirschmann-Jax C, Foster AE, Wulf GG, Nuchtern JG, Jax TW (2004). A distinct “side population” of cells with high drug efflux capacity in human tumor cells.. Proc Natl Acad Sci U S A.

[10] Buchakjian MR, Kornbluth S (2010). The engine driving the ship: metabolic steering of cell proliferation and death.. Nat Rev Mol Cell Biol.

[11] Kondoh H (2008). Cellular life span and the Warburg effect.. Exp Cell Res.

[12] Cairns RA, Harris IS, Mak TW (2011). Regulation of cancer cell metabolism.. Nat Rev Cancer.

[13] Gambhir SS (2002). Molecular imaging of cancer with positron emission tomography.. Nat Rev Cancer.

[14] Ortega AD, Sanchez-Arago M, Giner-Sanchez D, Sanchez-Cenizo L, Willers I (2009). Glucose avidity of carcinomas.. Cancer Lett.

[15] Denko NC (2008). Hypoxia, HIF1 and glucose metabolism in the solid tumour.. Nat Rev Cancer.

[16] Ding XW, Wu JH, Jiang CP (2010). ABCG2: a potential marker of stem cells and novel target in stem cell and cancer therapy.. Life Sci.

[17] Kitazoe K, Abe M, Hiasa M, Oda A, Amou H (2009). Valproic acid exerts anti-tumor as well as anti-angiogenic effects on myeloma.. Int J Hematol.

[18] Asano J, Nakano A, Oda A, Amou H, Hiasa M (2011). The serine/threonine kinase Pim-2 is a novel anti-apoptotic mediator in myeloma cells.. Leukemia.

[19] Hiasa M, Abe M, Nakano A, Oda A, Amou H (2009). GM-CSF and IL-4 induce dendritic cell differentiation and disrupt osteoclastogenesis through M-CSF receptor shedding by up-regulation of TNF-alpha converting enzyme (TACE).. Blood.

[20] Buda G, Maggini V, Galimberti S, Martino A, Giuliani N (2007). MDR1 polymorphism influences the outcome of multiple myeloma patients.. Br J Haematol.

[21] Turner JG, Gump JL, Zhang C, Cook JM, Marchion D (2006). ABCG2 expression, function, and promoter methylation in human multiple myeloma.. Blood.

[22] Allen JD, Brinkhuis RF, Wijnholds J, Schinkel AH (1999). The mouse Bcrp1/Mxr/Abcp gene: amplification and overexpression in cell lines selected for resistance to topotecan, mitoxantrone, or doxorubicin.. Cancer Res.

[23] Minderman H, O'Loughlin KL, Pendyala L, Baer MR (2004). VX-710 (biricodar) increases drug retention and enhances chemosensitivity in resistant cells overexpressing P-glycoprotein, multidrug resistance protein, and breast cancer resistance protein.. Clin Cancer Res.

[24] Modok S, Mellor HR, Callaghan R (2006). Modulation of multidrug resistance efflux pump activity to overcome chemoresistance in cancer.. Curr Opin Pharmacol.

[25] Szakacs G, Paterson JK, Ludwig JA, Booth-Genthe C, Gottesman MM (2006). Targeting multidrug resistance in cancer.. Nat Rev Drug Discov.

[26] Wu CP, Calcagno AM, Ambudkar SV (2008). Reversal of ABC drug transporter-mediated multidrug resistance in cancer cells: evaluation of current strategies.. Curr Mol Pharmacol.

[27] Mathupala SP, Ko YH, Pedersen PL (2009). Hexokinase-2 bound to mitochondria: cancer's stygian link to the “Warburg Effect” and a pivotal target for effective therapy.. Semin Cancer Biol.

[28] Pelicano H, Martin DS, Xu RH, Huang P (2006). Glycolysis inhibition for anticancer treatment.. Oncogene.

[29] Ko YH, Pedersen PL, Geschwind JF (2001). Glucose catabolism in the rabbit VX2 tumor model for liver cancer: characterization and targeting hexokinase.. Cancer Lett.

[30] Wolf A, Agnihotri S, Micallef J, Mukherjee J, Sabha N (2011). Hexokinase 2 is a key mediator of aerobic glycolysis and promotes tumor growth in human glioblastoma multiforme.. J Exp Med.

[31] Yalcin A, Telang S, Clem B, Chesney J (2009). Regulation of glucose metabolism by 6-phosphofructo-2-kinase/fructose-2,6-bisphosphatases in cancer.. Exp Mol Pathol.

[32] Mazurek S (2007). Pyruvate kinase type M2: a key regulator within the tumour metabolome and a tool for metabolic profiling of tumours.. Ernst Schering Found Symp Proc.

[33] Lin Q, Kim Y, Alarcon RM, Yun Z (2008). Oxygen and Cell Fate Decisions.. Gene Regul Syst Bio.

[34] Arai F, Suda T, Girard L (2008). Quiescent stem cells in the niche.. StemBook (Internet).

[35] Hulleman E, Kazemier KM, Holleman A, VanderWeele DJ, Rudin CM (2009). Inhibition of glycolysis modulates prednisolone resistance in acute lymphoblastic leukemia cells.. Blood.

