# Supplementary figures and images for: Glycolysis Inhibition Inactivates ABC Transporters to Restore Drug Sensitivity in Malignant Cells
Source: PLoS One. 2011 Nov 2;6(11):e27222. doi: 10.1371/journal.pone.0027222 (PMC3206937; doi:10.1371/journal.pone.0027222)

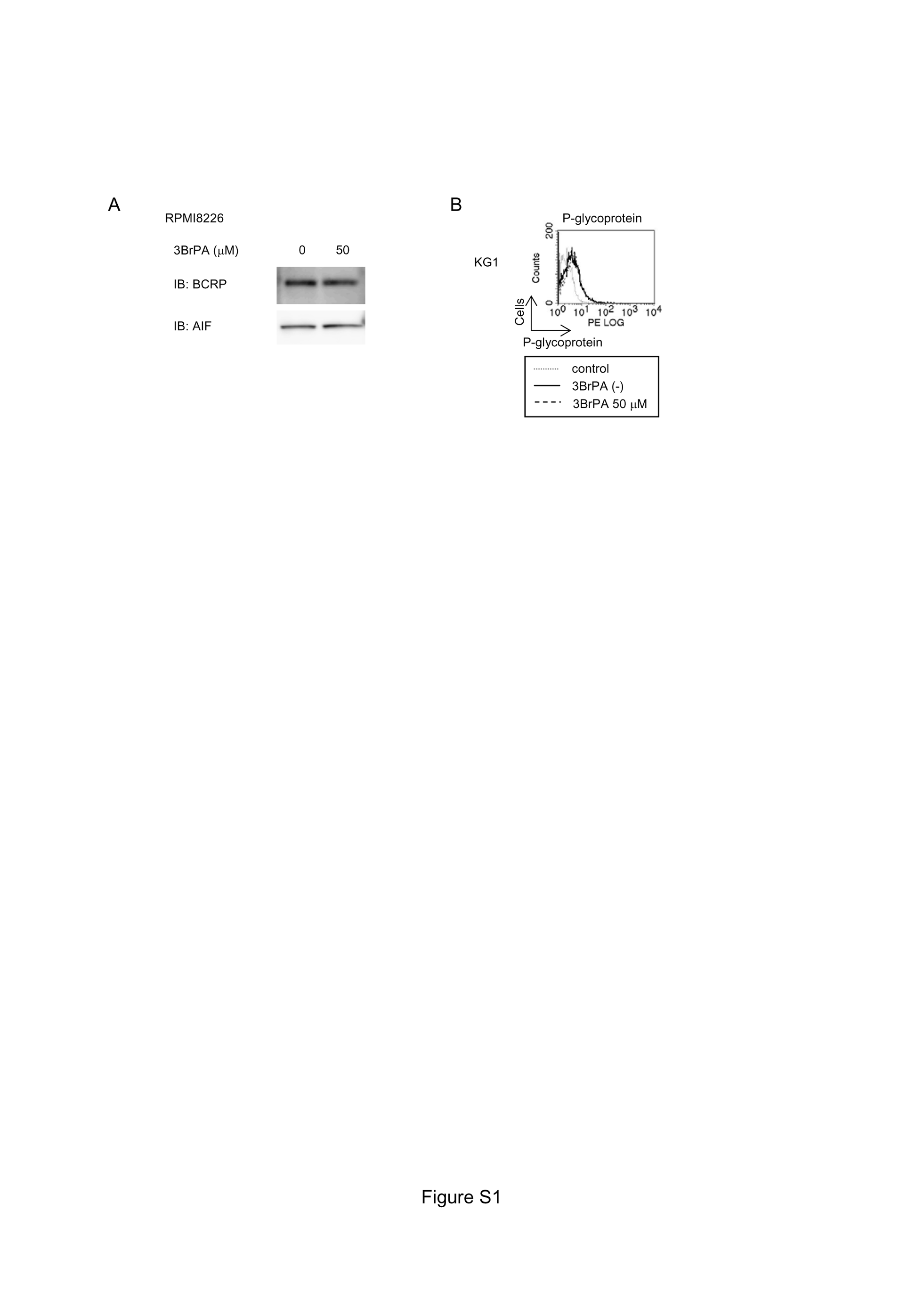

Supplement: Figure S1 — Surface membrane ABC transporter levels by glycolysis inhibition. A. RPMI8226 cells were cultured for 4 hours in the presence or absence of 3BrPA, and harvested. Membrane protein level of was analyzed by immunoblotting. B. Surface expression levels of P-glycoprotein in KG1 cells were analyzed by flow cytometry after culturing for 1 hour in the presence or absence of 3BrPA. (TIF) [file pone.0027222.s001.tif]

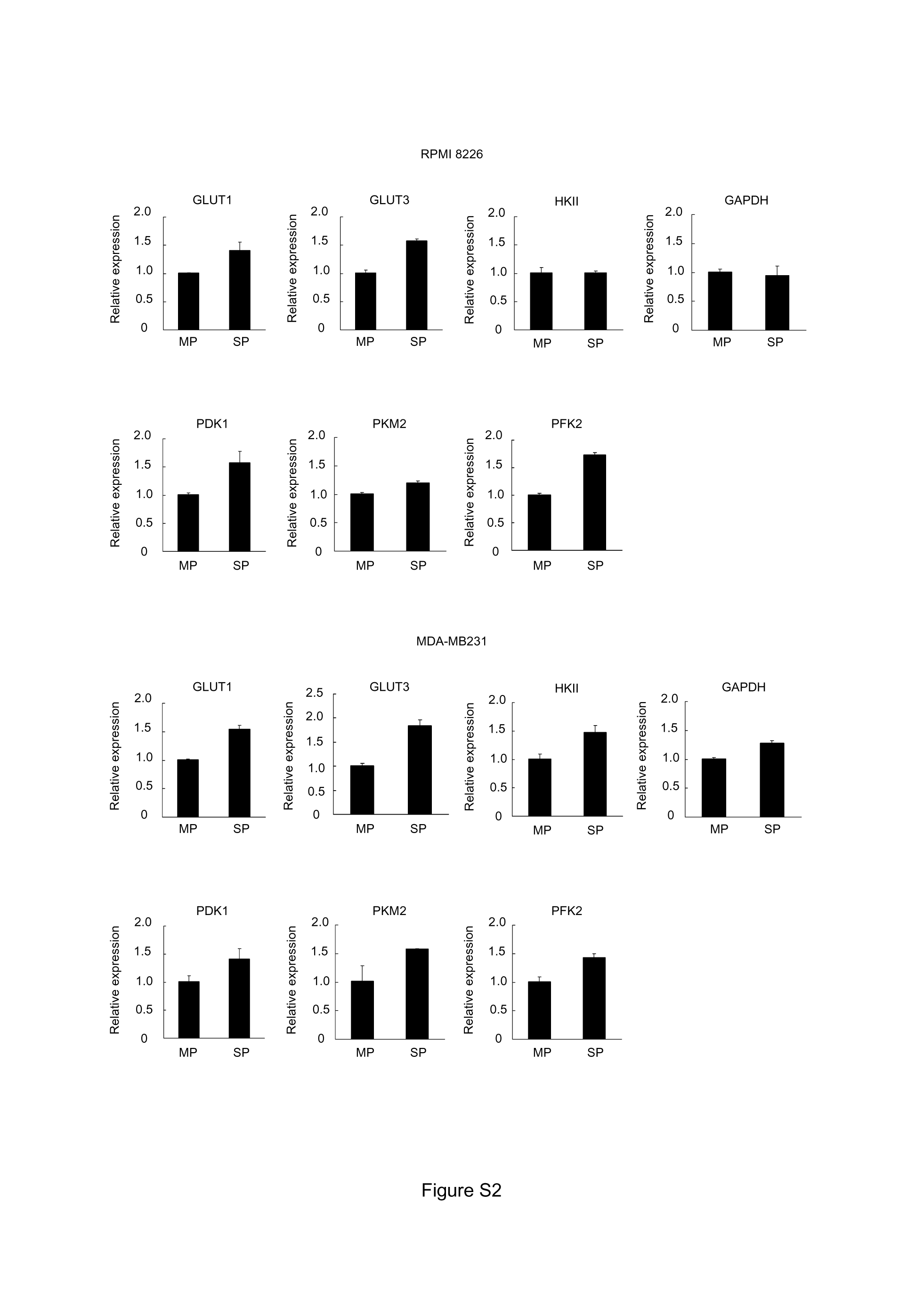

Supplement: Figure S2 — Glycolytic gene expression. Expression of genes involved in the glycolytic pathway were analyzed in SP and MP cells by quantitative real-time PCR. (TIF) [file pone.0027222.s002.tif]

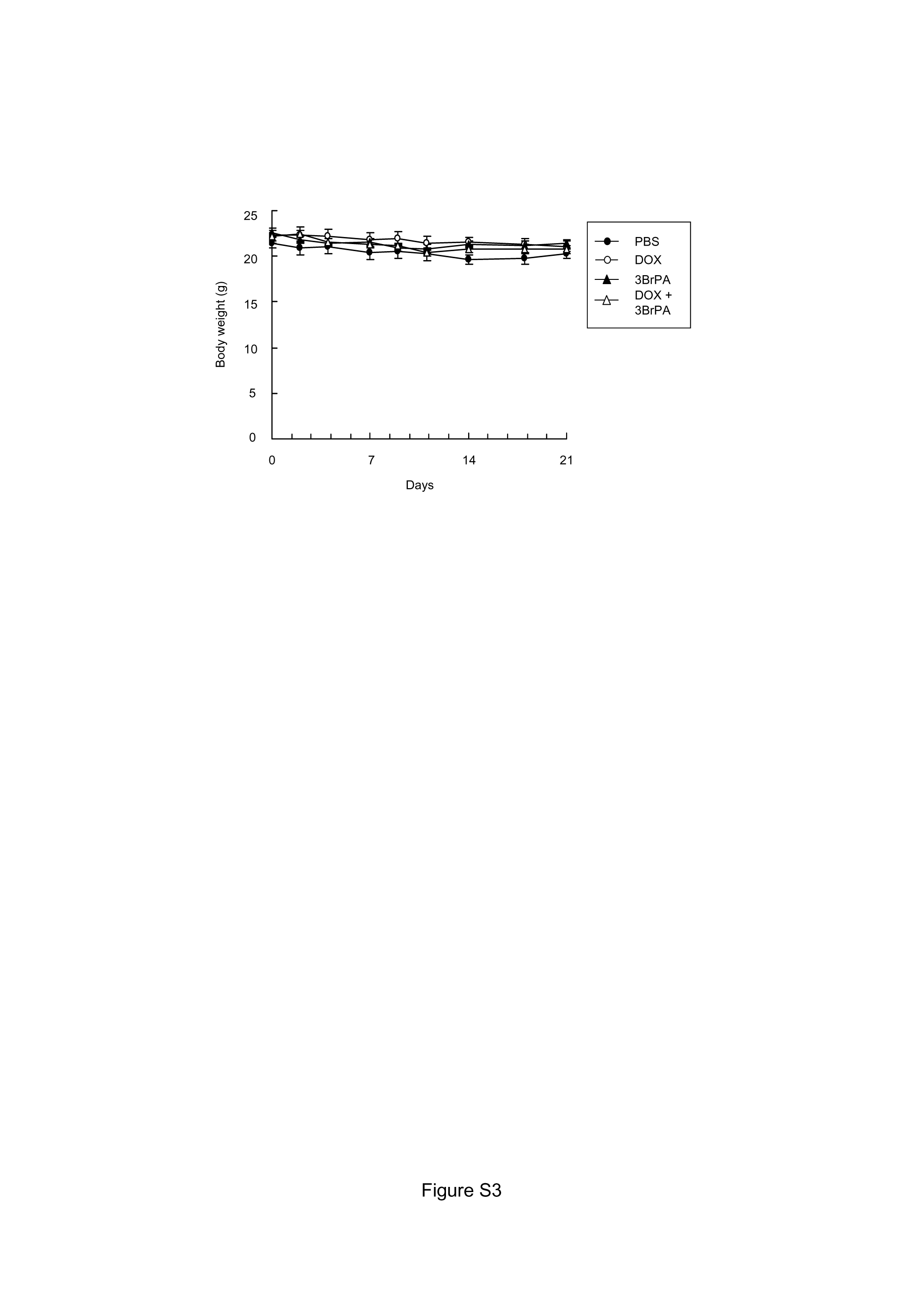

Supplement: Figure S3 — Body weight change. Body weights of the RPMI8226-bearing mice were measured at every treatment. (TIF) [file pone.0027222.s003.tif]
